# Esophageal variceal ligation in the secondary prevention of variceal bleeding: Result of long term follow-up

**DOI:** 10.11604/pamj.2013.15.3.2098

**Published:** 2013-05-03

**Authors:** Mounia Lahbabi, Ihssane Mellouki, Nouredine Aqodad, Mohammed Elabkari, Mounia Elyousfi, Sidi Adil Ibrahimi, Dafr Allah Benajah

**Affiliations:** 1 Department of Hepato Gastroenterology Hassan II University Hospital Fez, Morocco

**Keywords:** Variceal hemorrhage, endoscopic band ligation, liver cirrhosis, complication of band ligation, esophageal varices, secondary prevention

## Abstract

**Introduction:**

Long-term outcome of patients after band ligation have been poorly defined. Therefore, we conducted a long-term follow-up study to delineate the outcome of ligation in patients with portal hypertension in the Hassan II university hospital, Fes, Morocco.

**Methods:**

Over 118 months patients treated by endoscopic variceal ligation were received regular follow- up and detailed clinical assessment of at least 24 months.

**Results:**

One hundred twenty five patients were followed up for a mean of 31 months (range 12-107 months). Obliteration of the varices was achieved in 89.6 % (N = 112) of patients, with 3 +/-1.99 (range 1-8) endoscopy sessions over a period of 14 + /-6.8 weeks (range 3-28). The percentage of variceal recurrence during follow-up after ligation was 20.5 % (N = 23). Recurrence were observed in a mean of 22 months +/- 7.3 (range 3-48). Bleeding rate from recurrent varices was 30.4 % (7/23). Rebleeding from esophageal ulcers occurred in 5.6 % (7/125) of patients. Portal hypertensive gastropathy before and after eradication of varices was 17.6% (N = 22) and 44.6% (N = 50) respectively; p< 0.05. Fundal gastric varices was 30.4% (N = 38) and 35.7% (N = 40) before and after eradication of varices respectively; p> 0.05. The overall mortality was 4 % (N = 5).

**Conclusion:**

Band ligation was an effective technical approach for variceal obliteration with low rates of variceal recurrence, rebleeding and development of gastric varices. Furthermore, it was associated with frequent development of portal hypertensive gastropathy.

## Introduction

Approximately fifty-nine percent of patients with cirrhosis develop esophageal varices, and one-third of these patients experience esophageal variceal bleeding (EVB) [1]. There is considerable morbidity with EVB and the mortality rate with each episode is up to 30% [2, 3]. Recurrent bleeding is common without prophylactic treatment [4]; this risk varies between 8 and 35% within 2 years of follow up [5, 6], and an early rebleeding rate of 40-60% was noted within 7 and 10 days of a controlled index episode [2, 7]. This indicates that a correct therapeutic approach should be aimed not only at arresting the acute variceal bleeding episode but also at preventing an early variceal bleeding [8]. Thus, urgent treatment of the acute hemorrhage and steps to prevent rebleeding are essential. Endoscopic variceal ligation (EVL) has changed the outlook for patients with upper gastro intestinal bleeding. It is widely accepted as the optimum endoscopic treatment for esophageal variceal in the secondary prevention of EVB [9]. However, the rebleeding course and long-term outcome of patients with EVB after ligation have been poorly defined. Therefore, the aim of this retrospective study was to delineate the long term outcome of band ligation in patients with portal hypertension and who have already bled from their varices and treated by EVL.

## Methods

### Patients

This retrospective study was conducted in Endoscopy Unit of Department of Gastroenterology and Hepatology of Hassan II University Hospital Fez, Morocco, from December 2001 to October 2011. All adult patients with an episode of upper gastrointestinal bleeding (hematemesis or melena or both) were admitted to the emergency service, and underwent endoscopy as soon as they had been resuscitated. Patients with endoscopically proven esophageal variceal hemorrhage were treated by endoscopic variceal ligation if they had active variceal bleeding at endoscopy (spurting or oozing from esophageal varices); or if they had non bleeding varices but evidence of blood with no other potential source of gastrointestinal bleeding. Patients who were followed-up for at least 24 months were enrolled in the study. A full clinical history, physical examination, laboratory tests and ultrasonography were performed. Propranolol was initiated. The dose was increased stepwise, every two-three days, up to the maximum tolerated dose or up to 160 mg/day. The severity of liver disease was classified according to Child′s classification. The esophageal varices gradings were made as follows: 1= disappeared with air insufflation; 2= did not disappear with air insufflation; 3= occluded more than one-third of esophageal lumen [10].

### Endoscopic ligation

Endoscopies were performed in a single endoscopy unit using an Olympus video endoscope GIF 160. Esophageal varices were classified according to uniform criteria. Fundal varices and portal hypertensive gastropathy were also noted. After local application of lidocaine, an endoscope was introduced, and the ligation was carried out by placing a multiple rubber band (Saeed's multiband ligator Wilson's Cook) over a varice. Application of the bands was started at the gastro-esophageal junction and progressed upward in a helical way for approximately 5-8 cm. Patients underwent regular EVL until varices eradication (disappearance of varices or being too small to be sucked in the banding device).

### Follow-up

Patients were followed up from December 2001 to October 2011. Endoscopic ligation was performed every three weeks until the varices were obliterated or were reduced to a size of grade 1. In the latter instance, it was impossible to apply more bands because of the small size of the varices. The presence of ulcers, esophagitis or strictures was noted on endoscopic examination. After the varices had been obliterated or reduced in size to grade 1, patients underwent endoscopy every three months until the end of follow-up. If varices recurred and became larger or grade 2 in size, repeated ligation to obliterate was done. Patients were advised to refrain from consuming alcohol and from taking non steroidal anti-inflammatory drugs.

Variceal rebleeding: Any patient who had overt upper gastrointestinal bleeding during the study was admitted to the hospital and underwent endoscopy of the upper gastrointestinal tract within 24 hours to determine the source of bleeding. Bleeding from esophageal varices was diagnosed if active bleeding or a clot was seen on endoscopy and if there was evidence of recent bleeding in a patient with an esophageal varice and no other visible mucosal lesion. Bleeding was considered to be caused by esophageal ulcers as a result of band ligation if there was active bleeding or if there was an adherent clot on the esophageal ulcer [10].

### Endpoints

The main endpoint of the study was bleeding from any source. Secondary endpoints were the number of banding sessions, the time to eradication (calculated from the first banding session), the incidence of variceal recurrence, variceal rebleeding, complications and mortality.

### Statistical analysis

Data were analyzed by the Epi Info 2000. Quantitative data were expressed as means (± SD) or as medians.

### Ethics

This study was approved by the local Scientific Ethics Committee.

## Results

One hundred twenty five eligible patients were included in the study. They were followed up for a mean of 31 months (range 12-107 months). Fifty-eight (46.4%) were females, and sixty seven (53.6%) were males. Mean age of patients was 49.2 years (+/-7). Medical co morbidity associated with portal hypertension was noted in 11 patients (8.8%). Diabetes (8 cases), arterial hypertension (3 cases). Portal hypertension was due to cirrhosis in 67.2 % of cases (N = 84). The cause of cirrhosis was hepatitis C (HCV) alone 39,2 % (N = 33), hepatitis B (HBV) alone 9.5% (N = 8), combined HCV and HBV 4.7% (N = 4), alcohol 1.2 % (N = 1). Sixty nine (82.2 %) belonged to Child class A, twelve (14.3%) to child class B, and three patients (3.5%) were in Child class C. Propranolol was prescribed in association with esophageal variceal ligation as a secondary prophylaxis in 40.8 % of patients (N = 51). The dosage was variable according to the patient (until the heart rate had fallen by 25%). The characteristics of the patients are shown in Table1. High grade varices were seen in 67 patients (53.6%) while low grade varices were noted in 58 patients (46.4%). Obliteration of the varices was achieved in 89.6% (N = 112) of patients; with 3 +/-1.99 (range 1-8) endoscopy sessions; over a period of 14 + /-6.8 weeks (range 3-28). The percentage of variceal recurrence during follow-up was 20.5% (N = 23). Recurrence was observed in a mean of 22 months (range 3-48). Bleeding rate from recurrent varices was 30.4% (7/23). Rebleeding from EVL induced ulcers occurred in 5.6% (7/125) of ligation sessions. Portal hypertensive gastropathy before and after eradication of varices was 17.6% (N = 22) and 44.6% (N = 50) respectively; p < 0.05. The overall mortality was 4% (N = 5). One death (0.8%) were bleeding-related; the other causes of death were spontaneous ascites infection (2 cases), liver failure (2 cases). The results of endoscopic band ligation are shown in Table 2.

## Discussion

Endoscopic variceal ligation (EVL) is a purely mechanical method of obliterating varices that was introduced to preclude the undesirable effects of sclerotherapy [11]. Several studies have shown that EVL is effective and safe, requires few sessions to obliterate varices, and significantly reduces the rate of recurrent bleeding [12–14]. In the present study, EVL achieved variceal obliteration in 89.6% of patients with 3 +/-1.99 endoscopy sessions over a period of 14 + /-6.8 weeks. These findings were similar to the literature. A meta-analysis by Ko SY and others studies indicated that EVL achieved a variceal obliteration rates between 79% and 100% [15–18]. It has been shown previously that varices can be obliterated after 4-5 endoscopic sessions given over a period of 12-24 weeks [16, 17, 19].

Variceal recurrence during this period of study occurred in 20.5% (N = 23) of patients. While those found by others studies ranged between 8% and 48% after banding and 2% and 50% after sclerotherapy[20–25]. Interpretation of these results is complicated by the different lengths of follow-up in the studies and by differences in the definitions of variceal recurrence. At any rate, in recent years several combinations of endoscopic treatments have been proposed to reduce variceal recurrence after band ligation, but the clinical value of this combined treatments is still unknown [26–30].

During the study period, bleeding rate from recurrent varices was 30.4% (7/23). Others studies found rates ranged between 10% and 50% [31–33]. A relatively wide variation in rates of recurrent bleeding may be due, at least in part, to technical differences among studies, such as variations in the interval between sessions or in the number of bands placed during each session [34]. Whether these or other technical differences can affect the outcome has not been adequately investigated. Other possible confounding factors, such as the time since the initial bleeding episode, alcohol use or non-use, and the treatment used to stop the bleeding, may also affect the results of treatment. Among different trials, there may be differences in the characteristics of the population treated, such as the cause or the severity of hypertension, or in the definition of end points such as recurrent bleeding [35–40].

During the study period, the incidence of bleeding from treatment-induced ulcers was 5.6%. In others published studies, variceal ulcers have been reported between 3.3% and 36% [41, 42]. However, to our knowledge, most of these studies interested patient who underwent EVL for hemostasis of acute variceal bleeding. There is few data correlating the indication of EVL to the bleeding rate of ligation ulcers. One study recently published showed that bleeding from ligation ulcers occurred in 7.1% of cases after emergency ligation and in 0.5% of cases after electively performed banding ligation [43].

In this study, band ligation influences the development of portal hypertensive gastropathy (17.6% versus 44.6% before and after EVL respectively; p< 0.05). Some studies have shown that EVL results in an increase in the incidence and worsening of portal hypertensive gastropathy [44–47]. Others studies have shown that EVL influences also the development of gastric varices [48–50]. We in our study, contrary to these finding, haven't objectified the same results. A possible explanation on the key role of EVL in the development of Hypertensive gastropathy may be based on alteration of hemodynamics in patients with cirrhosis [51]. Kanke K et al concluded that cirrhotic gastric mucosa is in congestive condition [52], and EVL makes it more congestive soon after the procedure [53]. In addition, Korula J et al concluded that after EVL there is an increase in portal pressure gradient [54] which is transformed in worsening of portal hypertensive gastropathy and development of fundal varices [46].

In this study we were confronted with some limitations. First, our study is retrospective which make it difficult to compare to other studies generally prospective. Second, portal hypertensive gastropathy wasn't classified using the Baveno scoring system to classify its severity. And finally, the follow up time for these patients had varied from one year to ten years.

## Conclusion

In our population, endoscopic variceal ligation was an effective technical approach for variceal obliteration with low rates of variceal recurrence, rebeeding and development of fundal gastric varices. Furthermore, it was associated with frequent development of portal hypertensive gastropathy.
